# Supplementary material for: Cold-activated brown fat-derived extracellular vesicle-miR-378a-3p stimulates hepatic gluconeogenesis in male mice
Source: Nat Commun. 2023 Sep 6;14:5480. doi: 10.1038/s41467-023-41160-6 (PMC10482845; doi:10.1038/s41467-023-41160-6)
Supplement: Supplementary file 1 — Supplementary Information [file 41467_2023_41160_MOESM1_ESM.pdf]

SUPPLEMENTAL INFORMATION

Cold-activated brown fat-derived extracellular vesicle-miR-378a-3p stimulates hepatic gluconeogenesis in male mice

File list

1. Supplementary Figure S1

2. Supplementary Figure S2

3. Supplementary Figure S3

4. Supplementary Figure S4

5. Supplementary Figure S5

6. Supplementary Figure S6

7. Supplementary Figure S7

8. Supplementary Figure S8

9. Supplementary Table 1

10. Supplementary Table 2

11. Supplementary Table 3

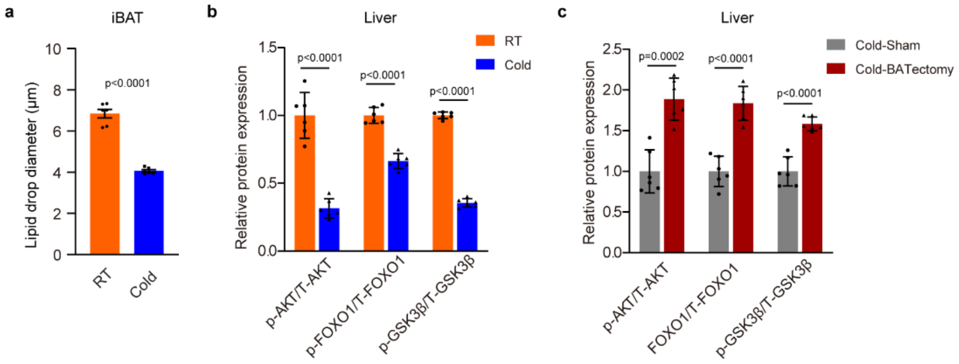

Supplemental Figure S1. Cold-activated BAT promotes hepatic gluconeogenesis, related to Figure 1. **a** Quantification of 1c, the diameter of the lipid droplets of iBAT (n=6 each group, from 2 independent experiments). **b** Quantification of bands of Western blotting, related to Figure 1j (n=6 each group, from 2 independent experiments). **c** Quantification of bands of Western blotting, related to Figure 1p (n=6 each group, from 2 independent experiments). Data presented as mean ± s.e.m. All statistical analysis was performed using two-sided unpaired t-test. Source data are provided as a Source Data file.

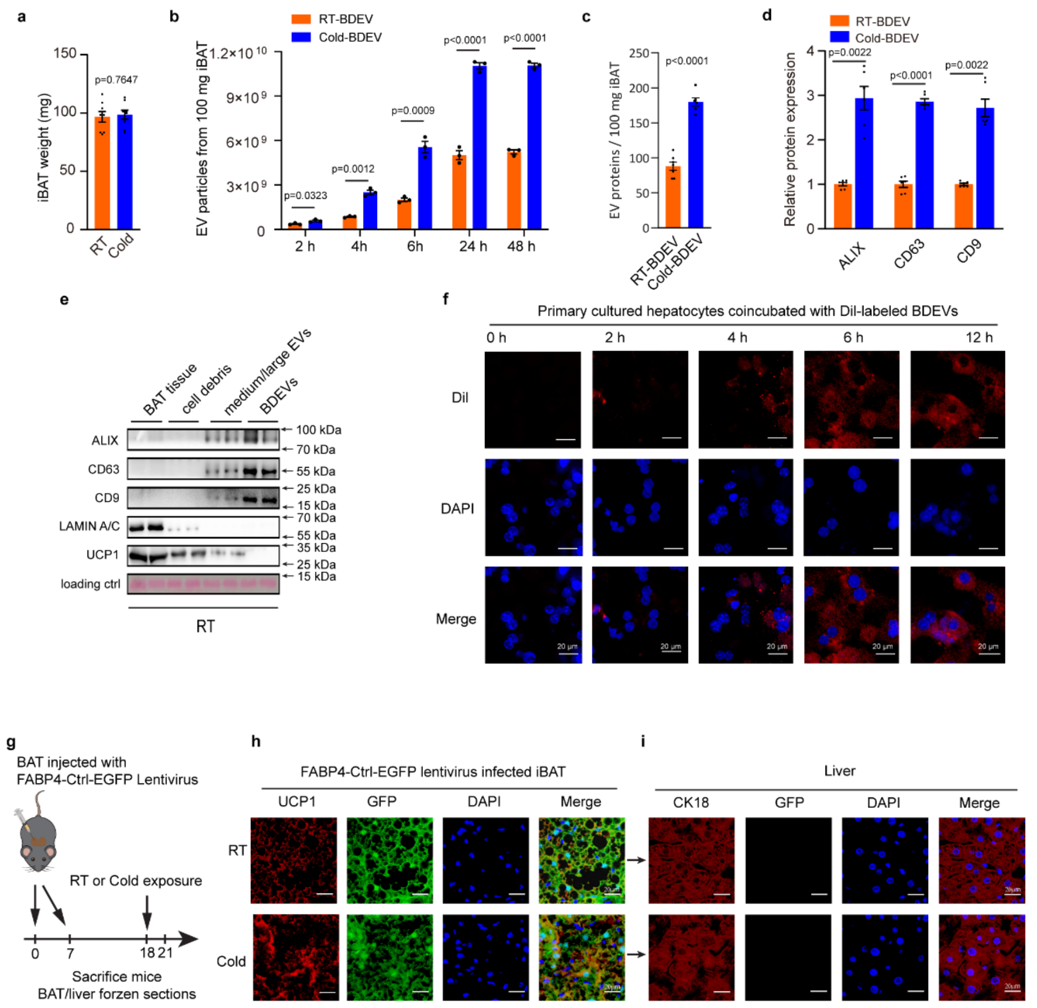

Supplemental Figure S2. Cold exposure stimulates EV secretion from BAT, related to Figure 2. a iBAT weight of RT- and Cold-exposed mice. (n=8 each group, from 2 independent experiment). **b** After 2 h, 6 h, 12 h, 24 h and 48 h of incubation, BDEVs particles in the culture medium of iBAT (n=3 each group). **c** Quantification of RT-BDEVs and Cold-BDEVs from 100 mg iBAT, related to Figure 2d (n=7,6, from 2 independent experiment). **d** Quantification of bands of western blotting detection of EV markers ALIX, CD63 and CD9 of the RT-BDEV and Cold-BDEV isolated from 100 mg iBAT, related to Figure 2e (n=6 each group, from 2 independent experiments). **e** Western blotting detection of the EV markers (ALIX, CD63, CD9), nuclear protein LAMIN A/C and mitochondrial inner membrane protein UCP1 in the iBAT, cell debris, medium/large EVs and BDEVs. (n=6 each group, from 2 independent experiment). **f** Confocal microscopy image of primary cultured hepatocytes coincubation with fluorescent Dil-labelled BDEVs for indicated time points. Scale bar: 20 μm. (n=3 each group, 5 random fields per sample, representative images were shown). **g-i** Experimental schematic procedure of *in vivo* Ctrl-EGFP labeled BDEV tracking. **(g)** Experimental schematic **(h, i)** Representative confocal microscopy image of coimmunostaining and GFP signal (green) in the iBAT (UCP1, red) **(h)** and the liver (CK18, red) **(i)** of mice infected with Ctrl-EGFP. (n=3 each group, 5 random fields per sample, representative images were shown). Scale bar: 20 μm. Data presented as mean ± s.e.m. Statistical analysis was performed two tailed Mann Whitney test (d) and others performed two-sided unpaired t-test. Source data are provided as a Source Data file.

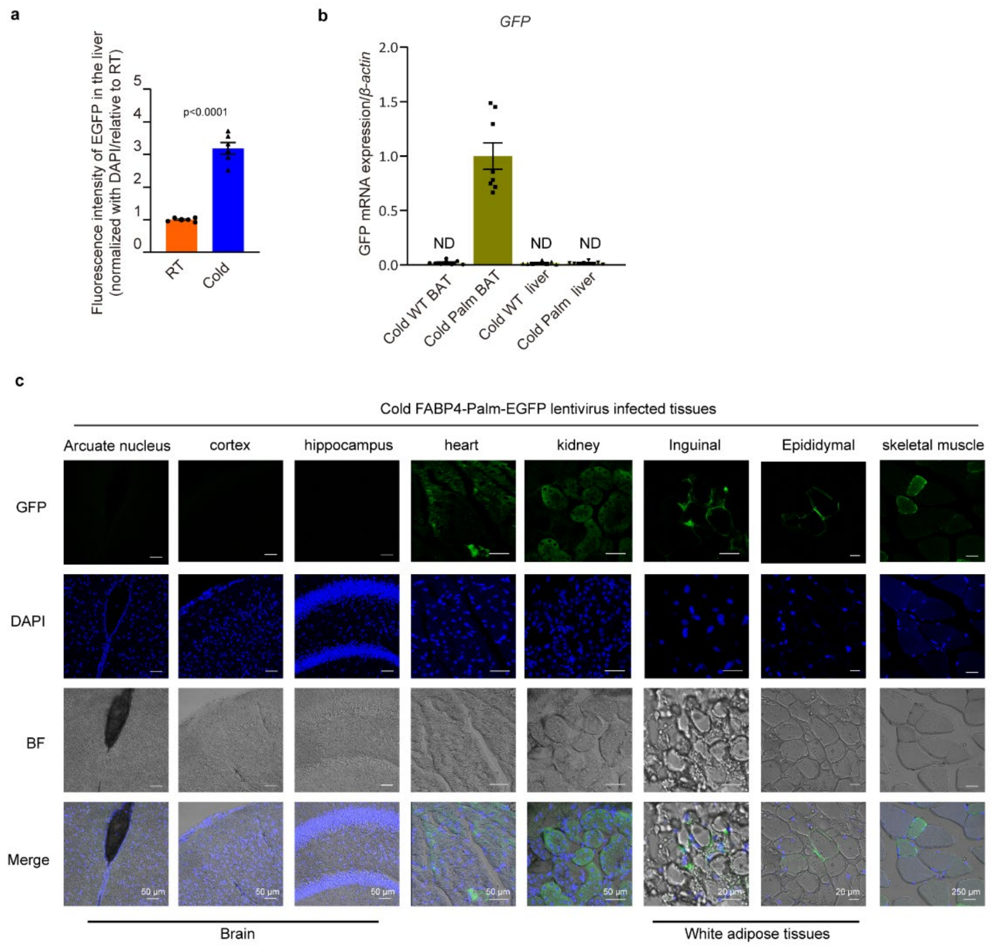

Supplemental Figure S3. Cold-BDEVs targeted tissues, related to Figure 2. a Quantification analysis of GFP fluorescence intensity in the liver from Palm-EGFP infected mice, related to Figure 2k (n=6 biological independent animals from 2 independent experiments). **b** GFP mRNA expression in the iBAT and liver after FABP4-Palm-EGFP lentivirus transduction. (n=8 biological replicates, from 2 independent experiments). **c** Representative confocal microscopy image of GFP signal (green) in the brain (arcuate nucleus of hypothalamus, hippocampus and cortex), kidney, heart, white adipose tissue (inguinal and epididymal adipose tissues) and skeletal muscle of mice infected with Palm-EGFP (n=3 each group, 5 random fields per sample, representative images were shown). Data presented as mean ± s.e.m. Statistical analysis was performed using two-sided unpaired t-test (a). Source data are provided as a Source Data file.

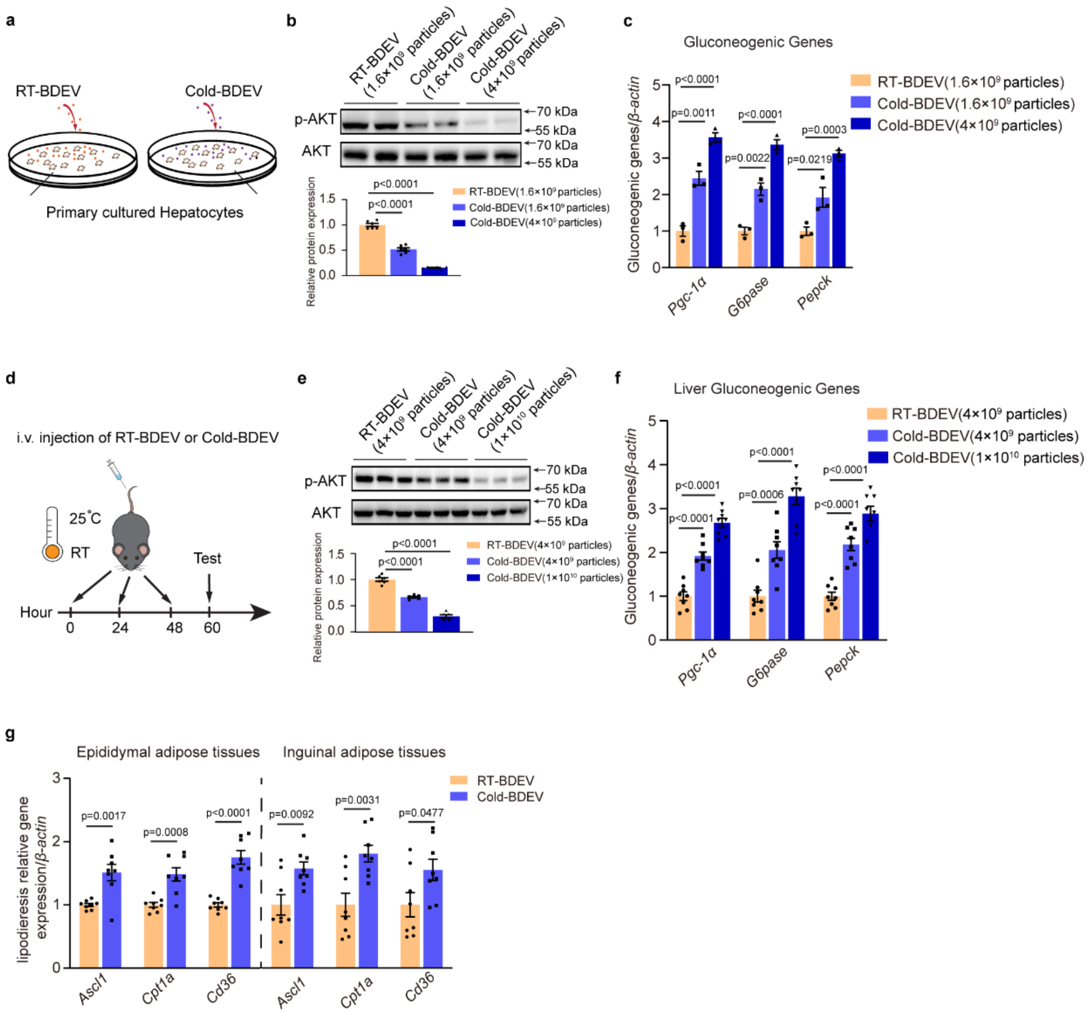

Supplemental Figure S4. Cold-BDEV induces gluconeogenesis, related to Figure 3. a-c 4 × 10^6 primary cultured hepatocytes were cocultured with 1.6×10^9 particles RT-BDEVs and equal Cold-BDEV particles (1.6×10^9) or from equal amounts of cultured Cold-BAT (4×10^9) for 48 hours. **(a)** Schematic diagram. **(b)** Western blotting and densitometry analysis of phosphorylated and total AKT in hepatocytes treated with RT-BDEVs or Cold-BDEVs (n=6 biological replicates, from 2 independent experiments). **(c)** Relative mRNA expression of gluconeogenic genes in hepatocytes treated with RT-BDEVs or Cold-BDEVs (n=3 biological replicates). **d-f** Eight-week-old male C57BL6/J mice were administered equal Cold-BDEV particles (4×10^9) or from equal amounts of Cold-BAT (1×10^{10}) once every day for a total of 3 injections, and subsequent studies were performed 12 hours after the last injection. **(d)** Schematic diagram. (**e**) Western blotting detection and densitometry analysis of p-AKT in the liver. (n=6 each group, from 2 independent experiment). **(f)** Relative mRNA expression of gluconeogenic genes in the liver (n=8 each group, from 2 independent experiments). **g** Relative mRNA expression of fatty acid oxidation genes *Cpt1a, Cd36* and *Acsl1* in the white adipose tissue (inguinal and epididymal adipose tissues) from mice treated with 80μg equal protein of RT-BDEVs or Cold-BDEVs (n=8 each group, from 2 independent experiments). Mice were fasted for 4 h before sacrifice. Data presented as mean ± s.e.m. Statistical analysis was performed two-sided unpaired t-test (g) and others performed one-way ANOVA followed by Bonferroni's multiple comparisons test. Source data are provided as a Source Data file.

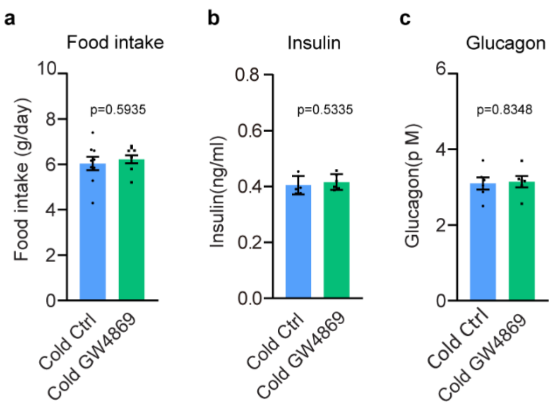

Supplemental Figure S5. Cold-BDEV induces gluconeogenesis, related to Figure 3. a-c Cold-exposed mice were injected *in situ* with either GW4869 or control vehicle at 0, 24 and 48 hours (for a total of 3 injections), mice were fasted 4 h before sacrifice. Food intake **(a**, n=9 each group, from 3 independent experiment), serum insulin (**b,** n=6 each group, from 2 independent experiment**)** and glucagon (**c,** n=6 each group, from 2 independent experiments) of cold-exposed mice treated with vehicle or GW4869. Data presented as mean ± s.e.m. All statistical analysis was performed using two-sided unpaired t-test. Source data are provided as a Source Data file.

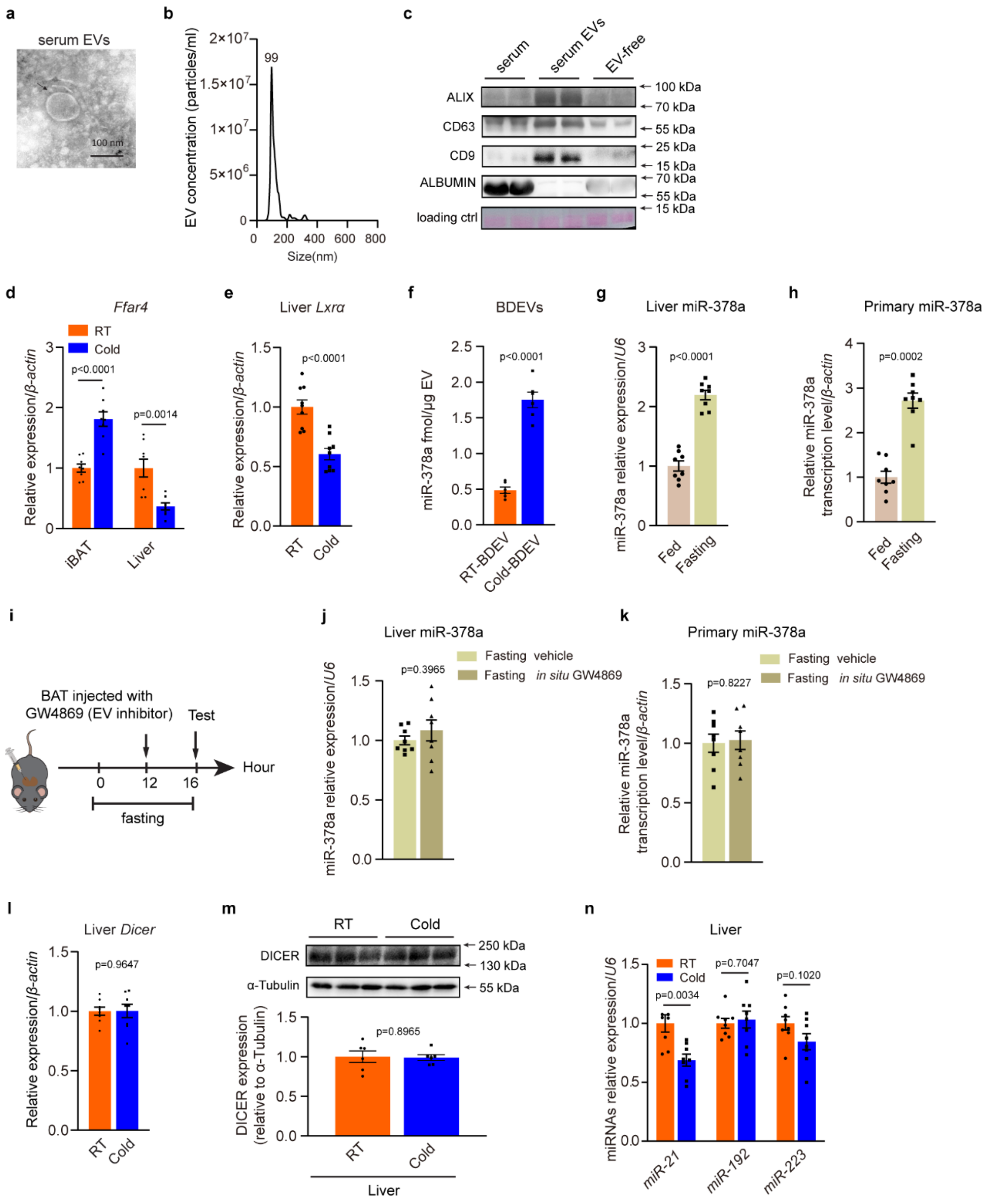

Supplemental Figure S6. Cold exposure increased miR-378a-3p expression in BDEVs, related to Figure 4. a-c Mice serum extracellular vesicle (serum EVs) identification. **(a)** TEM image of serum EVs. Scale bar: 100 nm. (n=3 each group, 5 random fields per sample, representative images were shown). **(b)** NTA of serum EVs (n=6 each group, from 2 independent experiments). **(c)** Western blotting detection of the EV markers (ALIX, CD63 and CD9) and negative protein marker ALBUMIN in the serum, serum EVs and EV-free serum (n=6 each group, from 2 independent experiments). **d** qPCR analysis of the relative levels of *Ffar4* in the iBAT and liver (n=8 each group, from 2 independent experiments). **e** qPCR analysis of the relative levels of *Lxrα* in the liver of RT or cold-exposed mice (n=9 each group, from 2 independent experiments). **f** The absolute level of miR-378a in the RT-BDEVs or Cold-BDEVs (n=6 each group, from 2 independent experiments). **g-h** Eight-week-old male C57BL6/J mice

were fasted overnight. (n=8 each group, from 2 independent experiments). qPCR analysis of the relative levels of miR-378a-3p **(g)** and primary miR-378a-3p **(h)** in the liver. **i-k** The fasted mice were injected in situ with GW4869 4 h prior to sacrifice. (n=8 each group, from 2 independent experiments). Schematic diagram **(i)**; qPCR analysis of the relative levels of miR-378a-3p **(j)** and primary miR-378a-3p **(k)** in the liver. **l-n** Eight-week-old male C57BL6/J mice were placed in RT (25 °C) or cold (4 °C) for 72 hours and fasted for 4 hours before sacrificed. **(l)** qPCR analysis of the relative levels of *Dicer* in the liver of RT or cold-exposed mice (n=8 each group, from 2 independent experiments). **(m)** Western blotting and densitometry analysis of DICER protein (n=6 each group, from 2 independent experiments). **(n)** qPCR analysis of the relative levels of miRNAs in the liver of RT or cold exposure mice (n=8 each group, from 2 independent experiments). Data presented as mean ± s.e.m. Statistical analysis was performed two tailed Mann Whitney test (h) and others performed two-sided unpaired t-test. Source data are provided as a Source Data file.

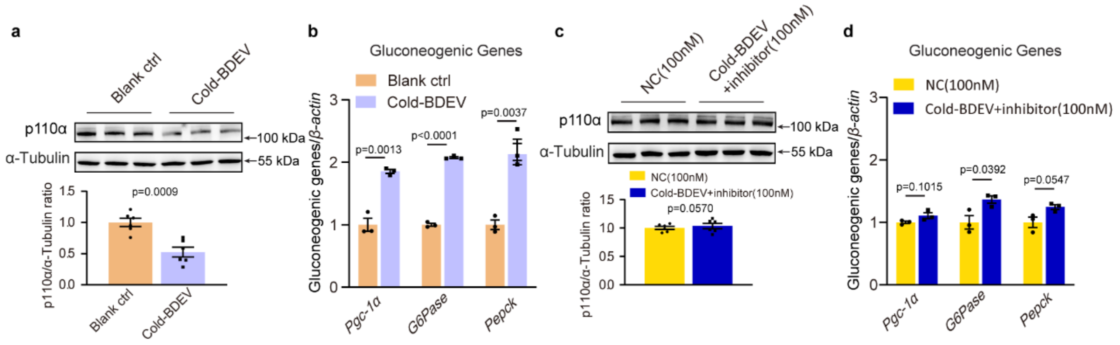

Supplemental Figure S7. The contribution of miR-378a-3p to Cold-BDEVs related to Figure 5. a-b 4 × 10^6 primary hepatocytes were co-cultured with or without 32 μg cold-BDEVs for 48 h. **(a)** Western blotting detection and densitometry analysis of p110α in hepatocytes. (n=6 each group, from 2 independent experiment); **(b)** Relative mRNA expression of gluconeogenic genes in hepatocytes (n=3 biological replicates). **c-d** 4 × 10^6 hepatocytes were transfected with 100 nM anti-miR-378a and co-culture with/without 32 μg cold-BDEVs for 48 h. **(c)** Western blotting detection and densitometry analysis of p110α in hepatocytes. (n=6 each group, from 2 independent experiment); **(d)** Relative mRNA expression of gluconeogenic genes in hepatocytes (n=3 biological replicates). Data presented as mean ± s.e.m. All statistical analysis was performed using two-sided unpaired t-test. Source data are provided as a Source Data file.

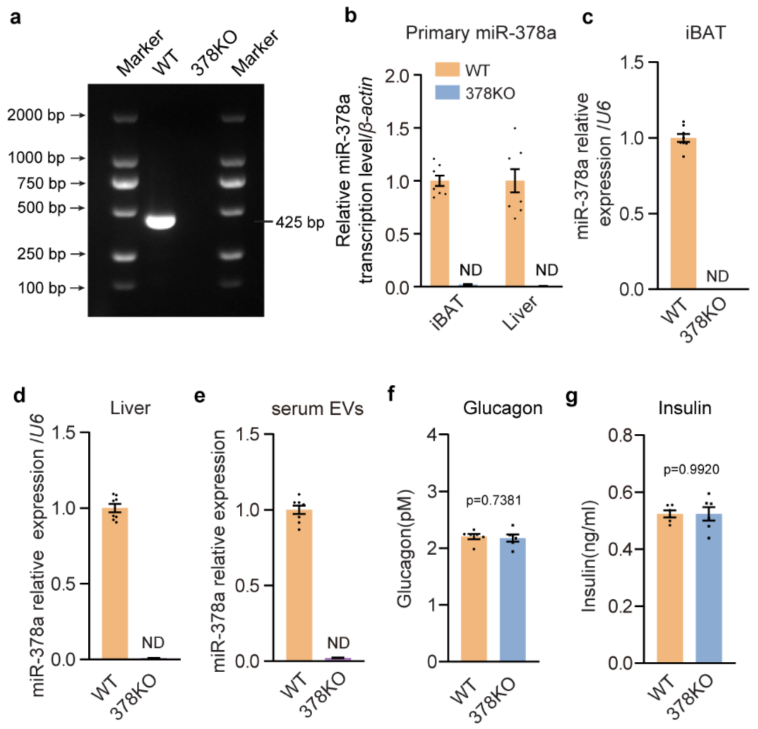

Supplemental Figure S8. Characterization of 378KO mice, related to Figure 7. a PCR image of genotyping of 378KO mice. **b-g** Eight-week-old male miR-378 KO mice and control WT littermates housed at RT. The mice were fasted for 4 hours before the analysis of metabolic parameters. **(b)** qPCR analysis of the relative miR-378a-3p transcription level in the iBAT and liver. **(c-e)** qPCR analysis of relative expression level of miR-378a in the iBAT **(c)**, liver **(d)** and serum EVs **(e)** of WT or miR-378 KO mice. (n=8 each group, from 2 independent experiments). **(f, g)** Serum glucagon **(f)** and insulin level **(g)**. (n=6 for each group, from 2 independent experiments). Data presented as mean ± s.e.m. All statistical analysis was performed using two-sided unpaired t-test. Source data are provided as a Source Data file.

Supplementary Tables

Supplementary Table 1. Nucleotide sequences of primers used for genotyping

Gene	Forward primer sequence (5' - 3')	Reverse primer sequence (5' - 3')
JS11882-miR-378a -wt	GGTTCCAAAGACTCTGTGCTGAC	TGCATGGCCTTTGGAGTCAAG
JS01882-Mir378a-wt	CACTTGCTGCCGTACTTTCACG	AAGATGGCTCCTACCAAAGGTAGC

Supplementary Table 2. Nucleotide sequences of primers used for qPCR

Gene	Species	Forward primer sequence (5' - 3')	Reverse primer sequence (5' - 3')
β-actin	Mouse	GGCTGTATTCCCCTCCATCG	CCAGTTGGTAACAATGCCATGT
Ucp1	Mouse	ACTGCCACACCTCCAGTCATT	CTTTGCCTCACTCAGGATTGG
Dio2	Mouse	CAGTGTGGTGCACGTCTCCAATC	TGAACCAAAGTTGACCACCAG
Elovl6	Mouse	TGCTGCATCCAGTTGAAGAC	TGCCATGTTCATCACCTTGT
Pgc-1α	Mouse	CCCTGCCATTGTTAAGACC	TGCTGCTGTTCCTGTTTTC
Pepck	Mouse	GTGCTGGAGTGGATGTTCGG	CTGGCTGATTCTCTGTTTCAGG
G6Pase	Mouse	AAGCCAACGTATGGATTCCG	ACAGCAATGCCTGACAAGACT
Pri-miR-378a-3p	Mouse	GCCGTGTTTCTCCATCTGTC	AGGGTCTGGGAAGGGCTGTG
Cpt1a	Mouse	GCTCGCACATTACAAGGACAT	TGGACACCACATAGAGGCAG
Cd36	Mouse	ATGGGCTGTGATCGGAACTG	GTCTTCCCAATAAGCATGTCTCC
Acsl1	Mouse	TGGGGTGGAAATCATCAGCC	CACAGCATTACACACTGTACAACGG
Ffar4	Mouse	ACCAAGTCAATCGCACCCAC	GTGAGACGACAAAGATGAGCC
Lxrα	Mouse	CTCAATGCCTGATGTTTCTCCT	TCCAACCCTATCCCTAAAGCAA
Gfp	Exogenous	AGTGCTTCGCCCGCTAC	CACCTTGATGCCGTTCTT
Dicer	Mouse	GAATAAGGCTTATCTTCTGCAGG	CATAAAGGTGCTTGGTTATGAGG

Supplementary Table 3. Antibodies Information

Target	Ventor	Catalogue No.	Working concentration	Validation	
				Reactivity	Application
UCP1	Cell Signaling Technology	72298	WB (1:1000), IF (1:100)	M R	WB, IHC, IF
α-Tubulin	Cell Signaling Technology	3873	WB (1:1000)	H M R Mk	WB, IHC, IF, F
p-PKM2	Cell Signaling Technology	3827	WB (1:1000)	H M R Mk	WB
PKM2	Cell Signaling Technology	4053	WB (1:1000)	H M R Mk	WB, IP, IF, F, IHC
p-AKT	Cell Signaling Technology	4060	WB (1:1000)	H M R Hm Mk Dm Z B	WB, IP, IF, F, IHC
AKT	Cell Signaling Technology	9272	WB (1:1000)	H M R Hm Mk C Dm B Dg Pg GP	WB, IP, IF, F
p-GSK3β	Cell Signaling Technology	9323	WB (1:1000)	H M R Mk	WB, IF, IHC
GSK3β	Cell Signaling Technology	12456	WB (1:1000)	H M R Mk	WB, IP, IF, F, IHC
p-FOXO1	Cell Signaling Technology	84192	WB (1:1000)	H M R Mk	WB, IP
FOXO1	Cell Signaling Technology	2880	WB (1:1000)	H M R Mk	WB, IP, IF, F, IHC, ChIP
p-110α	Cell Signaling Technology	4249	WB (1:1000)	H M R B	WB, IP
p-IR	Cell Signaling Technology	3024	WB (1:1000)	H M R	WB
IR	Cell Signaling Technology	3020	WB (1:1000)	H M R Mk	WB, IP
GLUT4	Millipore	07-1404	WB (1:1000)	H M	WB, IHC
PGC-1β	Santa Cruz Biotechnology	sc-373771	WB (1:1000)	M R	WB, IF, IP, ELISA
CD81	Abcam	ab109201	WB (1:1000)	H M R	WB
ALIX	Cell Signaling Technology	2171	WB (1:1000)	H M R Mk	WB, IP
CD9	Cell Signaling Technology	98327	WB (1:1000)	M R	WB, IF, F
CD63	Santa Cruz Biotechnology	sc5275	WB (1:1000)	H M R	WB, IP, IF, IHC, F, ELISA
DICER	Abcam	ab14601	WB (1:1000)	H M	WB, ChIP, F
ALBUMIN	Abcam	ab207327	WB (1:2000)	H M R	WB, IP, IF, F
LAMIN A/C	ABclonal	A19524	WB (1:10000)	H M R	WB, IP, IF, IHC
CK18	ABclonal	A19778	IF (1:100)	H M R	WB, IF, IHC
Horse anti-mouse IgG	Cell Signaling Technology	7076	WB (1:1000)	M	WB
Goat anti-rabbit IgG	Cell Signaling Technology	7074	WB (1:1000)	Rab	WB
Donkey anti-Rabbit Alexa 594	Invitrogen	A-21207	IF (1:1000)	Rab	IF, IHC, F
H-Human; M-Mouse R-Rat Hm-Hamster Mk-Monkey C-Chicken Dm-D. melanogaster Z-Zebrafish B-Bovine Dg-Dog Pg-Pig GP-Guinea Pig Rab-Rabbit					
WB-Western Blot IP-Immunoprecipitation IHC-Immunohistochemistry ChIP-Chromatin Immunoprecipitation IF-Immunofluorescence F-Flow Cytometry					
