# Supplementary material for: Cold-activated brown fat-derived extracellular vesicle-miR-378a-3p stimulates hepatic gluconeogenesis in male mice
Source: Nat Commun. 2023 Sep 6;14:5480. doi: 10.1038/s41467-023-41160-6 (PMC10482845; doi:10.1038/s41467-023-41160-6)
Supplement: Supplementary file 3 — Reporting Summary [file 41467_2023_41160_MOESM3_ESM.pdf]

nature portfolio

Corresponding author(s): Xiaohong Jiang

Last updated by author(s): Aug 17, 2023

Reporting Summary

Nature Portfolio wishes to improve the reproducibility of the work that we publish. This form provides structure for consistency and transparency in reporting. For further information on Nature Portfolio policies, see our Editorial Policies and the Editorial Policy Checklist.

Statistics

For all statistical analyses, confirm that the following items are present in the figure legend, table legend, main text, or Methods section.

n/a	Confirmed	
☐	☒	The exact sample size (*n*) for each experimental group/condition, given as a discrete number and unit of measurement
☐	☒	A statement on whether measurements were taken from distinct samples or whether the same sample was measured repeatedly
☐	☒	The statistical test(s) used AND whether they are one- or two-sided<br>*Only common tests should be described solely by name; describe more complex techniques in the Methods section.*
☒	☐	A description of all covariates tested
☐	☒	A description of any assumptions or corrections, such as tests of normality and adjustment for multiple comparisons
☐	☒	A full description of the statistical parameters including central tendency (e.g. means) or other basic estimates (e.g. regression coefficient) AND variation (e.g. standard deviation) or associated estimates of uncertainty (e.g. confidence intervals)
☐	☒	For null hypothesis testing, the test statistic (e.g. *F*, *t*, *r*) with confidence intervals, effect sizes, degrees of freedom and *P* value noted<br>*Give P values as exact values whenever suitable.*
☒	☐	For Bayesian analysis, information on the choice of priors and Markov chain Monte Carlo settings
☒	☐	For hierarchical and complex designs, identification of the appropriate level for tests and full reporting of outcomes
☒	☐	Estimates of effect sizes (e.g. Cohen's *d*, Pearson's *r*), indicating how they were calculated

Our web collection on statistics for biologists contains articles on many of the points above.

Software and code

Policy information about availability of computer code

Data collection	All fluorescence images were captured on a confocal microscope (Zeiss LSM880) with ZEN 3.6 blue edition software. Sections of H&E staining were captured under a virtual slide microscope (VS120, Olympus) with VS-ASW software(Olympus). Extracellular vesicles data were collected by NanoSight NS300(Malvern Instruments) through NanoSight NTA 3.2 software(Malvern Instruments). Quantitative real-time PCR software LC480 was used to collect real-time RCR data.
Data analysis	For quantification of western blots we used ImageJ-Fiji 2.0.0 software(NIH, Bethesda, MD).Histology staining and the fluorescence signals quantifications were performed using Image-Pro plus software 6.0 (Media Cybernetics).For statistical analyses, we used GraphPad PRISM 9 software .All data with significant difference were analyzed by GPower3.1 to obtain effect size and power value.

For manuscripts utilizing custom algorithms or software that are central to the research but not yet described in published literature, software must be made available to editors and reviewers. We strongly encourage code deposition in a community repository (e.g. GitHub). See the Nature Portfolio guidelines for submitting code & software for further information.

Data

Policy information about availability of data

All manuscripts must include a data availability statement. This statement should provide the following information, where applicable:

- Accession codes, unique identifiers, or web links for publicly available datasets
- A description of any restrictions on data availability
- For clinical datasets or third party data, please ensure that the statement adheres to our policy

All data generated in this study are provided in the Supplementary Information and Source data files. The miRNA microarray data generated by Trajkovski et al (GSE 41306) and by us (GSE 217222) are deposited at Gene Expression Omnibus repository. Source data are provided with this paper.

Research involving human participants, their data, or biological material

Policy information about studies with human participants or human data. See also policy information about sex, gender (identity/presentation), and sexual orientation and race, ethnicity and racism.

Reporting on sex and gender	N/A
Reporting on race, ethnicity, or other socially relevant groupings	N/A
Population characteristics	N/A
Recruitment	N/A
Ethics oversight	N/A

Note that full information on the approval of the study protocol must also be provided in the manuscript.

Field-specific reporting

Please select the one below that is the best fit for your research. If you are not sure, read the appropriate sections before making your selection.

☒ Life sciences ☐ Behavioural & social sciences ☐ Ecological, evolutionary & environmental sciences

For a reference copy of the document with all sections, see nature.com/documents/nr-reporting-summary-flat.pdf

Life sciences study design

All studies must disclose on these points even when the disclosure is negative.

Sample size	Sample sizes were indicated in the legend of each Figure and Supplementary Figure. No statistical tests were performed to pre-determine sample size. Sample sizes of all animal and cell studies were determined based on the basis of previous experiments in the lab and previous publications using similar methodologies (Mills et al. Nature, 2018, PMID30022159; Ding X. et al. Nature communications, 2021, PMID 33795678; Wang J. et al. Cell metabolism, 2022, PMID36070680) and a given power (>0.8) using G-power software 3.1. The sample sizes were sufficient for statistical analysis.
Data exclusions	No data were excluded from analysis.
Replication	Experiments were successfully replicated at least two to three times. The number of independent experiments and biological replicates in each data panel is indicated in the figure legends.
Randomization	Mice/cells were assigned randomly into experimental groups and processed in an arbitrary order. Age were matched between experimental groups.
Blinding	The investigators were not blind to allocation during experiments and outcome assessment,because the investigators need to conduct genotyping PCRs at the age of 2 weeks for the mice,therefore investigators were not blind for group identification or genotype identification for the mouse models used in this study. Blind was not relevant to the other experiments in cells because the investigators need to know what cell type they had to culture and process the cells by themselves.

Reporting for specific materials, systems and methods

We require information from authors about some types of materials, experimental systems and methods used in many studies. Here, indicate whether each material, system or method listed is relevant to your study. If you are not sure if a list item applies to your research, read the appropriate section before selecting a response.

Materials & experimental systems

n/a	Involved in the study
☐	☒ Antibodies
☐	☒ Eukaryotic cell lines
☒	☐ Palaeontology and archaeology
☐	☒ Animals and other organisms
☒	☐ Clinical data
☒	☐ Dual use research of concern
☒	☐ Plants

Methods

n/a	Involved in the study
☒	☐ ChIP-seq
☒	☐ Flow cytometry
☒	☐ MRI-based neuroimaging

Antibodies

Antibodies used

Following antibodies were used for western blot:
UCP1 antibody (Cell Signaling Technology, cat#72298, 1:1000)
α-Tubulin antibody (Cell Signaling Technology, cat#3873, 1:1000)
p-PKM2 antibody (Cell Signaling Technology, cat#3827, 1:1000)
PKM2 antibody (Cell Signaling Technology, cat#4053, 1:1000)
p-AKT antibody (Cell Signaling Technology, cat#4060, 1:1000)
AKT antibody (Cell Signaling Technology, cat#9272, 1:1000)
p-GSK3β antibody (Cell Signaling Technology, cat#9323, 1:1000)
GSK3β antibody (Cell Signaling Technology, cat#12456, 1:1000)
p-FOXO1 antibody (Cell Signaling Technology, cat#84192, 1:1000)
FOXO1 antibody (Cell Signaling Technology, cat#2880, 1:1000)
p-110α antibody (Cell Signaling Technology, cat#4249, 1:1000)
p-IR antibody (Cell Signaling Technology, cat#3024, 1:1000)
IR antibody (Cell Signaling Technology, cat#3020, 1:1000)
GLUT4 antibody (Millipore, cat#07-1404, 1:1000)
PGC-1β antibody (Santa Cruz Biotechnology, cat# sc-373771, 1:1000)
CD81 antibody (Abcam, cat#ab109201, 1:1000)
ALIX antibody (Cell Signaling Technology, cat#2171, 1:1000)
CD9 antibody (Cell Signaling Technology, cat#98327, 1:1000)
CD63 antibody (Santa Cruz Biotechnology, cat# sc5275, 1:1000)
DICER antibody (Abcam, cat#ab14601, 1:1000)
ALBUMIN antibody (Abcam, cat#ab207327, 1:2000)
LAMIN A/C antibody (ABclonal, cat#A19524, 1:10000)
Horse anti-mouse IgG (Cell Signaling Technology, cat#7076, 1:1000)
Goat anti-rabbit IgG (Cell Signaling Technology, cat#7074, 1:1000)
Following antibodies were used for immunofluorescence staining:
UCP1 antibody (Cell Signaling Technology, cat#72298, 1:100)
CK18 antibody (ABclonal, cat#A19778, 1:100)
Donkey anti-Rabbit IgG (H+L) Highly Cross-Adsorbed Secondary Antibody, Alexa Fluor™ 594 (Invitrogen, cat# A-21207, 1:1000)

Validation

All antibodies have been tested for reactivity against the appropriate species on the specification sheets on the providers' websites or in published articles.
Following antibodies were used for western blot:

UCP1 antibody is suitable for WB, IHC, IF and reacts with mouse and rat.
https://www.cellsignal.com/products/primary-antibodies/ucp1-e9z2v-xp-rabbit-mab/72298

α-Tubulin antibody is suitable for WB, IHC, IF, F and reacts with human, mouse, rat etc.
https://www.cellsignal.com/products/primary-antibodies/a-tubulin-dm1a-mouse-mab/3873

p-PKM2 antibody is suitable for WB and reacts with human, mouse, rat etc.
https://www.cellsignal.com/products/primary-antibodies/phospho-pkm2-tyr105-antibody/3827

PKM2 antibody is suitable for WB, IP, IF, F, IHC and reacts with human, mouse, rat etc.
https://www.cellsignal.com/products/primary-antibodies/pkm2-d78a4-xp-rabbit-mab/4053

p-AKT antibody is suitable for WB, IP, IF, F, IHC and reacts with human, mouse, rat etc.
https://www.cellsignal.com/products/primary-antibodies/phospho-akt-ser473-d9e-xp-rabbit-mab/4060

AKT antibody is suitable for WB, IP, IF, F and reacts with human, mouse, rat etc.
https://www.cellsignal.com/products/primary-antibodies/akt-antibody/9272

p-GSK3β antibody is suitable for WB, IF, IHC and reacts with human, mouse, rat etc.

https://www.cellsignal.com/products/primary-antibodies/phospho-gsk-3b-ser9-5b3-rabbit-mab/9323

GSK3β antibody is suitable for WB, IP, IF, F, IHC and reacts with human, mouse, rat etc.
https://www.cellsignal.com/products/primary-antibodies/gsk-3b-d5c5z-xp-rabbit-mab/12456

p-FOXO1 antibody is suitable for WB, IP and reacts with human, mouse, rat etc.
https://www.cellsignal.com/products/primary-antibodies/phospho-foxo1-ser256-e1f7t-rabbit-mab/84192

FOXO1 antibody is suitable for WB, IP, IF, F, IHC, ChIP and reacts with human, mouse, rat etc.
https://www.cellsignal.com/products/primary-antibodies/foxo1-c29h4-rabbit-mab/2880

p-110α antibody is suitable for WB, IP and reacts with human, mouse, rat etc.
https://www.cellsignal.com/products/primary-antibodies/pi3-kinase-p110a-c73f8-rabbit-mab/4249

p-IR antibody is suitable for WB and reacts with human, mouse, rat.
https://www.cellsignal.com/products/primary-antibodies/phospho-igf-i-receptor-b-tyr1135-1136-insulin-receptor-b-tyr1150-1151-19h7-rabbit-mab/3024

IR antibody is suitable for WB, IP and reacts with human, mouse, rat etc.
https://www.cellsignal.com/products/primary-antibodies/insulin-receptor-b-l55b10-mouse-mab/3020

GLUT4 antibody is suitable for WB, IHC and reacts with human, mouse.
https://www.sigmaaldrich.cn/CN/zh/product/mm/071404

PGC-1β antibody is suitable for WB, IF, IP, ELISA and reacts with mouse, rat etc.
https://www.scbt.com/p/pgc-1beta-antibody-e-9?requestFrom=search

CD81 antibody is suitable for WB and reacts with human, mouse, rat.
https://www.abcam.cn/cd81-antibody-epr4244-ab109201.html

ALIX antibody is suitable for WB, IP and reacts with human, mouse, rat etc.
https://www.cellsignal.com/products/primary-antibodies/alix-3a9-mouse-mab/2171

CD9 antibody is suitable for WB, IF, F and reacts with mouse, rat.
https://www.cellsignal.com/products/primary-antibodies/cd9-e8l5j-rabbit-mab/98327

CD63 antibody is suitable for WB, IP, IF, IHC, F, ELISA and reacts with human, mouse, rat.
https://www.scbt.com/p/cd63-antibody-mx-49-129-5?requestFrom=search

DICER antibody is suitable for WB, ChIP, F and reacts with human, mouse.
https://www.abcam.cn/products/primary-antibodies/dicer-antibody-13d6-chip-grade-ab14601.html

ALBUMIN antibody is suitable for WB, IP, IF, F and reacts with human, mouse, rat.
https://www.abcam.cn/products/primary-antibodies/albumin-antibody-epr20195-ab207327.html

LAMIN A/C antibody is suitable for WB, IP, IF, IHC and reacts with human, mouse, rat.
https://abclonal.com.cn/catalog/A19524

Horse anti-mouse IgG is suitable for WB and reacts with mouse.
https://www.cellsignal.com/products/secondary-antibodies/anti-mouse-igg-hrp-linked-antibody/7076

Goat anti-rabbit IgG is suitable for WB and reacts with rabbit.
https://www.cellsignal.com/products/secondary-antibodies/anti-rabbit-igg-hrp-linked-antibody/7074

Following antibodies were used for immunofluorescence staining:

UCP1 antibody is suitable for WB, IHC, IF and reacts with mouse and rat.
https://www.cellsignal.com/products/primary-antibodies/ucp1-e9z2v-xp-rabbit-mab/72298

CK18 antibody is suitable for WB, IF, IHC and reacts with human, mouse, rat.
https://abclonal.com.cn/catalog/A19778

Donkey anti-Rabbit Alexa Fluor™ 594 is suitable for IF, IHC, F and reacts with rabbit.
https://www.thermofisher.cn/cn/zh/antibody/product/Donkey-anti-Rabbit-IgG-H-L-Highly-Cross-Adsorbed-Secondary-Antibody-Polyclonal/A-21207

Eukaryotic cell lines

Policy information about cell lines and Sex and Gender in Research

Cell line source(s)	HEK 293T Cells were obtained from the American Type Culture Collection (ATCC No. CRL-11268) and were cultured at 37℃ and 5% CO2 in Dulbecco's modified Eagle's medium(DMEM) supplemented with 10% FBS，1，000U/mL penicillin and 100g/mL streptomycin.
Authentication	The cell line was not authenticated.
Mycoplasma contamination	Mycoplasma contamination was not tested in the study.
Commonly misidentified lines (See ICLAC register)	No commercial misidentified cells were used.

Animals and other research organisms

Policy information about studies involving animals; ARRIVE guidelines recommended for reporting animal research, and Sex and Gender in Research

Laboratory animals	All animal experimental procedures were conducted in accordance with the National Institutes of Health Guide for the Care and Use of Laboratory Animals and were approved by the Animal Ethical Board of Nanjing University (IACUC-2104002). To reduce the variations that may result from hormonal changes and avoid the possible influences that caused by the estrogen cycle on glucose metabolism in female mice41-43, only male C57BL/6 mice were used in the present study. Eight-week-old male C57BL/6J WT mice were from GemPharmatech Laboratory (Nanjing, China). The miR-378 KO mice were generated on a C57BL/6J background at the GemPharmatech Laboratory (Nanjing, China). We used CRISPR/Cas9 technology to modify miR-378a gene, exon1 of miR-378a-201 (ENSMUST00000198300.1) transcript is recommended as the knockout region. Primers used for genotyping are shown in Supplementary Table 1. Heterozygous 378KO mice were mated to produce littermate WT and KO mice for study. To calculate food intake, the experimental animals were raised individually in a specific pathogen free (SPF) facility at Nanjing University and maintained at 21±2°C and a relative humidity of 55±10% with free access to pellet normal chow diet (cat#SWS9102, XieTong Biology) and water and kept on a 12h light/12h dark cycle. For cold exposure, the mice were housed individually in a 4°C incubator for 72 h, and the mice were weighed before and after cold (Δ body weight).
Wild animals	none
Reporting on sex	Only male mice were used in the present study. In the study design, male mice were selected primarily based on the following considerations: 1）gender differences in glucose metabolism have been reported in mice due to the different expression pattern of sex hormones (Varlamov O et al., Front Endcrinol. 2015; Jan 19; 5:241. PMID 25646091). In particular, estrogen, which reaches a maximal (~60 pg/ml) in the proestrus stage and is reduced to ~20 pg/ml at estrus in female mice (Saito T et al., Circ Res. 2009 Aug 14; 105(4):343-352. PMID: 19608983), was shown to suppress gluconeogenesis (Yan H et al., Diabetes. 2019 Feb; 68(2):291-304.PMID: 30487265). Therefore, to avoid the possible influences caused by the estrogen cycle on glucose metabolism in female mice, male mice were chosen for the current study. Moreover, whether gender differences exist in response to cold exposure in mice is still controversial. According to a recent study on young adults, short-term cold exposure showed more pronounced hormonal changes in women than in men (Mengel L et al., J Clin Endocrinol Metab. 2020; May 105 (5): e1938-e1948. PMID:32144431). Therefore, to reduce the variations that may result from hormonal changes in female mice, only male mice were chosen for the current study. Our appreciation for the reviewer's emphasis on this critical issue. We have added considerations regarding to gender differences in the study design and indicated in the title and abstract that only male mice were used in the present study.
Field-collected samples	none
Ethics oversight	All experimental procedures were approved by Model Animal Research Center of Nanjing University and according with Laboratory Animal Care Guidelines.

Note that full information on the approval of the study protocol must also be provided in the manuscript.
